# Analysis of spatial-temporal distribution of notifiable respiratory infectious diseases in Shandong Province, China during 2005–2014

**DOI:** 10.1186/s12889-021-11627-6

**Published:** 2021-08-30

**Authors:** Xiaomei Li, Dongzhen Chen, Yan Zhang, Xiaojia Xue, Shengyang Zhang, Meng Chen, Xuena Liu, Guoyong Ding

**Affiliations:** 1 School of Public Health, Shandong First Medical University & Shandong Academy of Medical Sciences, No.619 Changcheng Road, Taian, 271016 Shandong Province China; 2 Liaocheng Center for Disease Control and Prevention, Liaocheng, 252100 Shandong Province China; 3 Guiqian International General Hospital, Guiyang, 550018 Guizhou Province China; 4 Qingdao Municipal Center for Disease Control & Prevention, Qingdao, 266033 Shandong Province China; 5 Shandong Center for Disease control and Prevention, Jinan, 250014 Shandong Province China; 6 Jining Center for Disease Control and Prevention, Qingdao, 272113 Shandong Province China

**Keywords:** Notifiable respiratory infectious diseases, Spatial-temporal distribution, Spatial autocorrelation, Spatial-temporal clustering

## Abstract

**Background:**

Little comprehensive information on overall epidemic trend of notifiable respiratory infectious diseases is available in Shandong Province, China. This study aimed to determine the spatiotemporal distribution and epidemic characteristics of notifiable respiratory infectious diseases.

**Methods:**

Time series was firstly performed to describe the temporal distribution feature of notifiable respiratory infectious diseases during 2005–2014 in Shandong Province. GIS Natural Breaks (Jenks) was applied to divide the average annual incidence of notifiable respiratory infectious diseases into five grades. Spatial empirical Bayesian smoothed risk maps and excess risk maps were further used to investigate spatial patterns of notifiable respiratory infectious diseases. Global and local Moran’s *I* statistics were used to measure the spatial autocorrelation. Spatial-temporal scanning was used to detect spatiotemporal clusters and identify high-risk locations.

**Results:**

A total of 537,506 cases of notifiable respiratory infectious diseases were reported in Shandong Province during 2005–2014. The morbidity of notifiable respiratory infectious diseases had obvious seasonality with high morbidity in winter and spring. Local Moran’s *I* analysis showed that there were 5, 23, 24, 4, 20, 8, 14, 10 and 7 high-risk counties determined for influenza A (H1N1), measles, tuberculosis, meningococcal meningitis, pertussis, scarlet fever, influenza, mumps and rubella, respectively. The spatial-temporal clustering analysis determined that the most likely cluster of influenza A (H1N1), measles, tuberculosis, meningococcal meningitis, pertussis, scarlet fever, influenza, mumps and rubella included 74, 66, 58, 56, 22, 64, 2, 75 and 56 counties, and the time frame was November 2009, March 2008, January 2007, February 2005, July 2007, December 2011, November 2009, June 2012 and May 2005, respectively.

**Conclusions:**

There were obvious spatiotemporal clusters of notifiable respiratory infectious diseases in Shandong during 2005–2014. More attention should be paid to the epidemiological and spatiotemporal characteristics of notifiable respiratory infectious diseases to establish new strategies for its control.

**Supplementary Information:**

The online version contains supplementary material available at 10.1186/s12889-021-11627-6.

## Background

Although the incidence of infectious diseases has been effectively controlled, infectious diseases are still a major global public health problem. At the global level, infectious diseases amount to an estimated 18% of the total disability adjusted life years (DALYs) of the Global Burden of Disease (GBD) in 2016 [1]. At present, infectious diseases still represent a significant public health problem in China, with over 10 million cases (incidence rate was 733.57 per 100,000) reported in 2019 [2]. Notifiable respiratory infectious diseases resulting from bacterial or viral pathogens such as *mycobacterium tuberculosis*, streptococcus pneumoniae, respiratory syncytial virus (RSV) or influenza virus are major global public health concerns. For example, pandemic of coronavirus disease 2019 (one of notifiable respiratory infectious diseases) led to 172.64 million cases and 3.72 million deaths worldwide by June 8, 2021 [3]. Being one of the provinces with high incidence rates of notifiable respiratory infections diseases in China, the annual incidence rate of notifiable respiratory infections diseases in Shandong Province ranged from 0.003 to 30.148 per 100,000 in the total population in 2017, with highest incidence of tuberculosis (30.148 per 100,000) and lowest incidence of meningococcal meningitis (0.003 per 100,000) [4].

One study suggested that the spread of disease varied significantly from place to place and from time to time for diverse causes [5]. Many studies have explored the distribution of spatial-temporal clusters in the epidemiology of a particular type of notifiable respiratory infectious diseases. For example, the spatial and temporal characteristics of influenza A (H7N9) was described in China using geographic information systems (GIS) method [6–8]. One study provided a quantitative description of the age-specific morbidity pandemic patterns of influenza A (H1N1) across administrative areas of Peru [9]. Several studies about the spatial-temporal distribution of tuberculosis such as Beijing [10], Yunnan [11, 12], Qinghai [13] and mainland of China [14] were analyzed in China. Based on GIS technology and spatial statistics, one study explored areas and periods at high risk of scarlet fever in China [15]. Another Chinese study was conducted to evaluate the significance of aggregation and determine the size of the range of hotspots at the county level in Guangxi [16]. These studies have shown the geographical and temporal heterogeneity of notifiable respiratory infectious diseases epidemic.

To our knowledge, only one study has systematically explored the epidemiologic trends and spatial changing patterns of notifiable respiratory infectious diseases, but which analyzed at the provincial level in China [17]. Therefore, cluster analysis of notifiable respiratory infectious diseases at a more precise level is urgently needed. With little research has been conducted in Shandong Province, China, the overall epidemic trend of notifiable respiratory infectious diseases remains unknown. This study aimed to detect the spatial-temporal clusters of notifiable respiratory infectious diseases at county-level of Shandong. Knowledge of spatial-temporal distribution of notifiable respiratory infectious diseases was crucial to understand the dynamic transmission of notifiable respiratory infectious diseases and to provide local evidence for prevention and control strategies of notifiable respiratory infectious diseases.

## Methods

### Study area

The study was conducted in Shandong Province (longitude 114°47.5′-122°42.3′E and latitude 34°22.9′-38°24.01′N), which is located in the lower reaches of Yellow River with Bohai Sea and Yellow Sea in the East (See Supplementary Fig. 1, Additional File 1). By 2020, Shandong Province has 16 prefecture-level cities (i.e., Jinan, Qingdao, Zibo, Zaozhuang, Dongying, Yantai, Weifang, Jining, Taian, Weihai, Rizhao, Binzhou, Liaocheng, Linyi, Dezhou and Heze), 57 municipal districts, 27 county-level cities, 53 counties and 137 county-level administrative regions in total. In 2018, the prefecture-level city of Laiwu was abolished, which assigned to Jinan. Therefore, Laiwu was still studied as a prefecture-level city for the study period was from 2005 to 2014. Shandong Province covers an area of 155,800 km^2^ and has a permanent resident population of 101.5 million. As of 2019, there were 84,000 medical institutions in Shandong Province, with 629,800 beds and 1009,100 working personnel. Among them, there were 2651 hospitals, 867 hospitals above Grade II and 80,000 community-level medical and health institutions, with a total of 675 million visits made by medical institution.

### Data source

Notifiable respiratory infectious diseases data for this study were derived from the China Information System for Disease Control and Prevention. All notifiable respiratory infectious diseases cases were defined base on the diagnostic criteria and principles of management for notifiable infection diseases issued by National Health Commission of the People’s Republic of China. Only the cases confirmed by both clinical and laboratory tests, including microscopic examination and biochemical identification, were included in the study. The number of cases of diphtheria and severe acute respiratory syndrome (SARS) was too low during the study period. Therefore, it was difficult to analyze the spatial-temporal clusters of diphtheria and SARS. Therefore, these two diseases were not included in our study. Demographic data of Shandong Province were collected from the Sixth National Population Census in 2010 and the Center for Public Health Science Data in China (http://www.phsciencedata.cn/).

### Study design and statistical analysis

Spatial, temporal and spatiotemporal analysis were performed to understand the temporal and spatial distribution characteristics of notifiable respiratory infectious diseases and determine its spatial-temporal clustering in Shandong Province. We examined the temporal and spatial distribution characteristics of notifiable respiratory infectious diseases in the following three-step process.

Firstly, Time series was applied to describe the temporal distribution characteristics of notifiable respiratory infectious diseases. Monthly incidence rate of each notifiable respiratory infectious disease during the study period (2005–2014) was exhibited to observe the temporal trends of each notifiable respiratory infectious disease. We used the ArcGIS 10.4 software to produce the thematic maps of the average annual incidence rate of each notifiable respiratory infectious disease. The average annual incidence rate was graded by Natural Break (Jenks), which was divided into 5 levels. The Natural Break (Jenks) can identify break points by picking the class breaks that best group similar values and maximize the differences between classes [18]. Since the onset of notifiable respiratory infectious diseases is a low-probability event with aggregation and instability in China, empirical Bayes can make statistical adjustment according to the principle that large population is more stable than small population, so as to obtain more stable clustering and make the results more accurate and reliable [19]. In our study, spatial empirical Bayesian smoothing was used to adjust the average annual incidence rate of each notifiable respiratory infectious disease, and the estimation rate based on spatial weight matrix was closer to the real situation of geographical distribution of diseases [19]. GeoDa 1.10 software was used to apply spatial empirical Bayesian smoothing and map the excess risk (ER) of each notifiable respiratory infectious disease.

Secondly, spatial autocorrelation analysis was used to identify the spatial clustering of each notifiable respiratory infectious disease in Shandong Province. Moran’s *I* is an important indicator to analyze the spatial distribution characteristics of disease cases. Global Moran’s *I* was used to detect whether the significant spatial autocorrelation regions of each notifiable respiratory infectious disease in Shandong Province existed [20]. Then local Moran’s *I* was further used to clarify the patterns of spatial autocorrelation in spatial units. The Moran’s *I* rangs from − 1 to 1, which indicates a positive correlation when the value is > 0 and a negative correlation when the value is < 0 at a statistically significant level. By using local indicators of spatial association (LISA) map [21, 22], four different spatial clustering modes were demonstrated, which were: High-High (HH, high-incidence regions surrounded by high-incidence regions, i.e., hotspots), Low-Low (LL, low-incidence regions surrounded by low-incidence regions, i.e., coldspots), High-Low (HL, high-incidence regions surrounded by low-incidence regions) and Low-High (LH, low-incidence regions surrounded by high-incidence regions). Spatial autocorrelation analysis was performed using the univariate (local) Moran’s *I* tool in the GeoDa 1.10 software. The significance level was set at *P* < 0.05 and 95% confidence interval (CI) with the number of simulations at 999.

Lastly, the space-time scan statistic was adopted to explore the spatial-temporal clusters of each notifiable respiratory infectious disease. In this study, the monthly incidence was taken as the clustering unit and the county as the minimum spatial unit. We used a cylindrical scanning window for probe scanning. The maximum spatial cluster size and maximum temporal cluster size were all set to 50%. For each window, the expected number of cases can be inferred by using the discrete Poisson model with the observed number of cases and the number of the population within/outside the moved windows (the potential clusters) of candidate regions during candidate time [23, 24]. The relative risk (RR) was calculated by the ratio of the observed number to the expected number within the windows and outside the windows. Log-likelihood ratio (LLR) was employed to identify the special clusters by comparing the observed incidence with expected one, and the *P* value of LLR was obtained by the Monte Carlo method with the simulation time 999 [23]. The window with the largest LLR value was defined as the most likely cluster, and other windows that contained clusters with statistically significant LLR values were defined as the first secondary cluster, the second secondary cluster, etc. The analysis process was carried out using the software SaTScan 9.4.4 and the scanning results were visualized by ArcGIS 10.4 software.

## Results

### Descriptive analysis for notifiable respiratory infectious diseases

There were 537,506 cases of notifiable respiratory infectious diseases in total in Shandong Province over the study period. Table 1 shows the incidence of each notifiable respiratory infectious disease in study area during the study period. Nine notifiable respiratory infectious disease types were reported during the study period, with annual average incidences ranging from 0.026 to 36.450 per 100,000.

**Table 1:** The incidence of each notifiable respiratory infectious disease in Shandong Province during the study period

Disease	Time-period	Number of cases	Average annual incidence rate (/10^5^)
Influenza A (H1N1)	2009.5–2014.12	4755	1.008
Measles	2005.1–2014.12	25,994	2.772
Tuberculosis	2005.1–2014.12	34,3161	36.450
Meningococcal meningitis	2005.1–2014.12	242	0.026
Pertussis	2005.1–2014.12	1606	0.170
Scarlet fever	2005.1–2014.12	22,554	2.366
Influenza	2005.1–2014.12	23,874	2.496
Mumps	2005.1–2014.12	105,305	11.065
Rubella	2005.1–2014.12	10,014	1.069

### The temporal distribution of notifiable respiratory infectious diseases

**Fig. 1:**
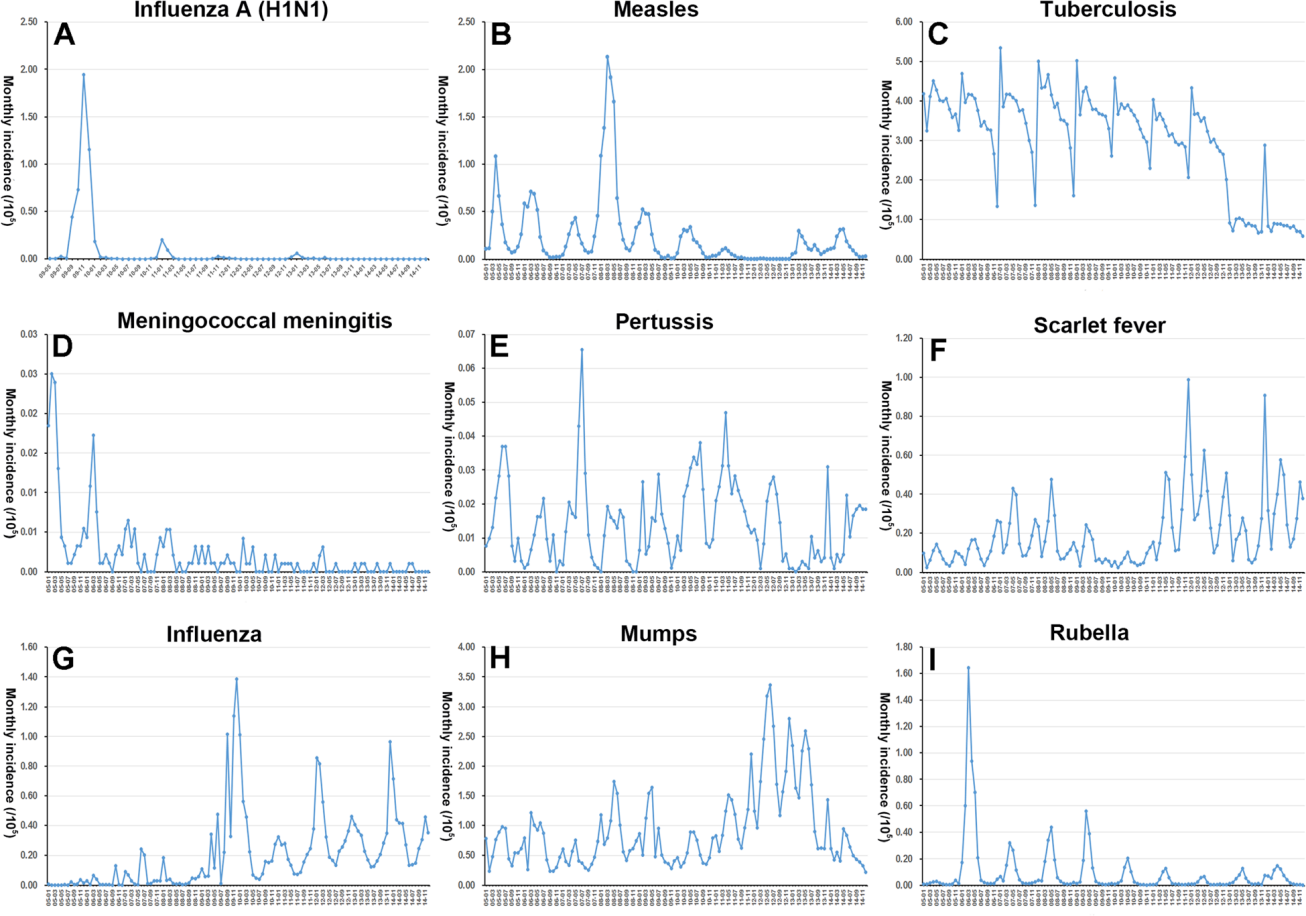
Monthly incidence of notifiable respiratory infectious diseases from 2005 to 2014 in Shandong Province

The monthly incidence of each notifiable respiratory infectious disease is shown in Fig. 1. The time series of influenza A (H1N1) indicated outbreaks of influenza A (H1N1) occurred in the winter of 2009, with the highest monthly incidence (1.96 per 100,000) in November 2009 and sporadic in other years (See Fig. 1A). The monthly incidence of measles had an obvious seasonal increase in spring and peaked in March 2008 (2.13 per 100,000). The incidence of measles dropped significantly after 2010 and then stabilized (See Fig. 1B). The monthly incidence rate of tuberculosis was between 4 per 100,000 and 5 per 100,000, which peaked in late winter and early spring, mainly from January to April. At the overall level, the peak of incidence of tuberculosis was observed in 2008, and then decreasing (See Fig. 1C). From 2005 to 2014, the monthly incidence of meningococcal meningitis showed a significant downward trend, with peaking in late spring, early summer (May–June) and winter (December, See Fig. 1D). The incidence of pertussis was stable and low during the study period (< 0.1 per 100,1000, See Fig. 1E). The monthly incidence of scarlet fever remained relatively stable in 2005–2009, but began to increase rapidly since 2010. The incidence of scarlet fever showed a bimodal seasonal pattern with a peak occurring in summer and a peak occurring in winter (See Fig. 1F). The incidence of influenza rapidly increased since 2009 with strong seasonality in winter, and its peak occurred in December 2009 (1.39 per 100,000, See Fig. 1G). The incidence of mumps was relatively stable during the study period with a fluctuation in 2012 (See Fig. 1H). The time series indicated outbreaks of rubella occurred in March 2006 with the monthly incidence rate of 1.65 per 100,000 (See Fig. 1I).

### The spatial distribution of notifiable respiratory infectious diseases

**Fig. 2:**
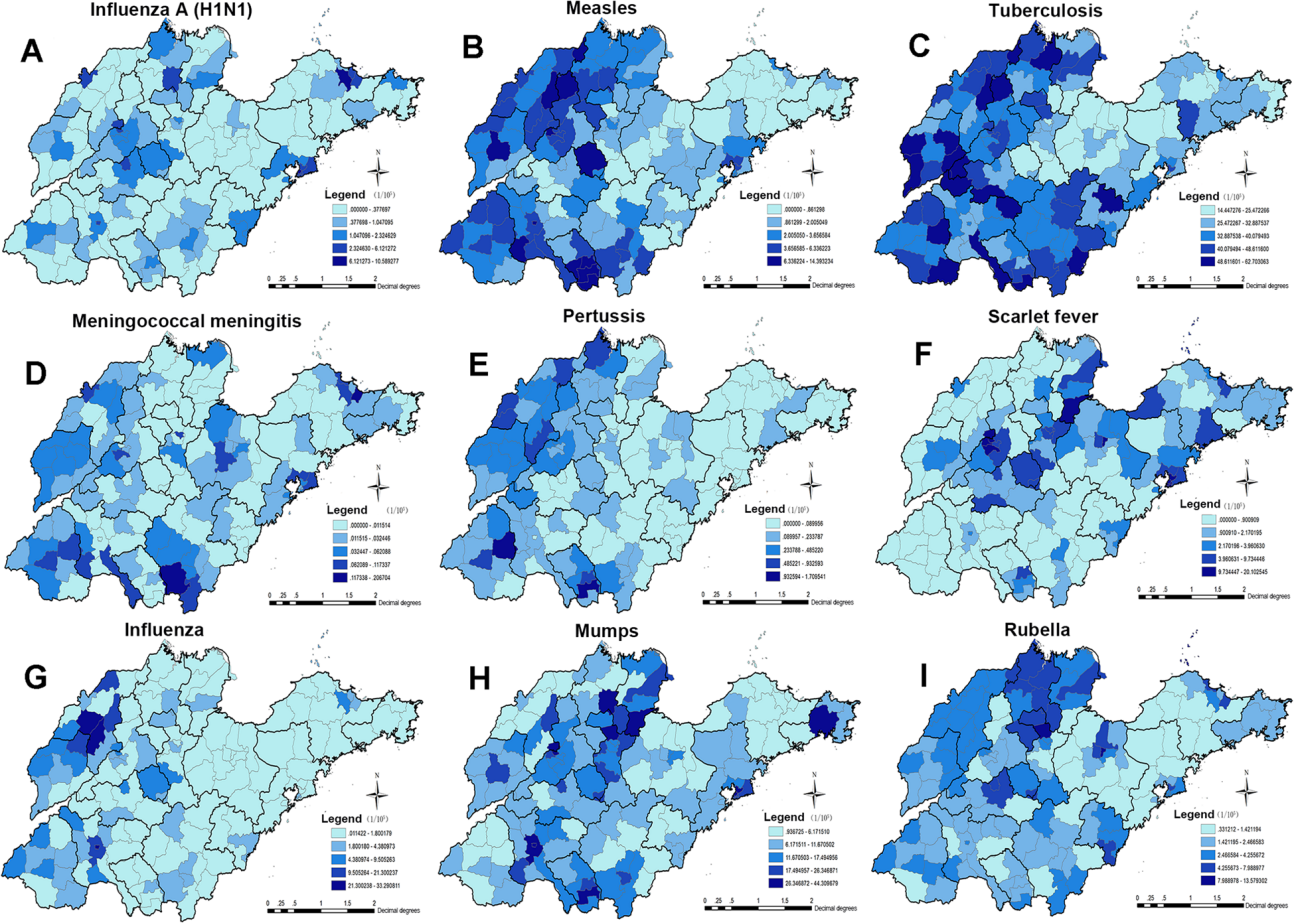
The average annual incidence rate of notifiable respiratory infectious diseases by the natural breaks (Jenks) method from 2005 to 2014 in Shandong Province. The Shandong map was created with ArcGIS software based on the public geographical data downloaded from Resource and Environment Science and Data Center, Institute of Geographic Sciences and Natural Resources Research, CAS (https://www.resdc.cn/)

The spatial distribution of notifiable respiratory infectious diseases in Shandong Province is shown in Fig. 2 and Fig. S2 (See Supplementary Fig. 2, Additional File 1). The incidence of influenza A (H1N1) was relatively dispersed, and the top three counties with the higher incidence were Sifang District in Qingdao (10.6 per 100,000), Fushan District in Yantai (8.6 per 100,000) and Taishan District in Taian (6.1 per 100,000, See Fig. 2A). After spatial empirical Bayesian smoothing, the highly incidence of influenza A (H1N1) occurred in Jinan and Northwest Shandong (See Supplementary Fig. 2A, Additional File 1). There were 13 counties in the incidence of influenza A (H1N1) with ER above 4, such as Wudi County and Huimin County in Dezhou, and there were 26 counties with ER greater than 1 and less than 4 (See Supplementary Fig. 3A, Additional File 1). The incidence of measles is high in Northwest and South Shandong with average annual incidence rate of 6.5 per 100,000 to 15.0 per 100,000 (See Fig. 2B). The result of measles after Bayesian smoothing was consistent with that of Natural Break classification (See Supplementary Fig. 2B, Additional File 1). There were 60 counties in the incidence of measles with ER greater than 1 (See Supplementary Fig. 3B, Additional File 1). We can see that tuberculosis struck all counties, with a higher incidence in West Shandong, Northwest Shandong and Southeast Shandong from the thematic map and Bayesian smoothing map of tuberculosis (See Fig. 2C & Supplementary Fig. 2C, Additional File 1). The highest annual incidence of tuberculosis was in Dongping County in Taian (62.7 per 100,000), followed by Qingyun County in Dezhou (60.6 per 100,000). ER above 1 covered 72 counties in the ER map of tuberculosis (See Supplementary Fig. 3C, Additional File 1). The incidence of meningococcal meningitis was relatively low in the whole Shandong, with a higher incidence in Liaocheng, Heze, Jining and Linyi (See Fig. 2D). After Bayesian smoothing, the incidence of meningococcal meningitis decreased in Northern and Central Shandong (See Supplementary Fig. 2D, Additional File 1). The ER map of meningococcal meningitis showed 55 counties with ER greater than 1 (See Supplementary Fig. 3D, Additional File 1). The incidence of pertussis was also relatively low in Shandong province, with a higher incidence in Northwestern Shandong and Southern Shandong (See Fig. 2E). After Bayesian smoothing, it showed that the incidence of pertussis decreased in Pingyuan County in Dezhou and increased in Central Shandong (See Supplementary Fig. 2E, Additional File 1). The ER of 49 counties was greater than 1 for pertussis (See Supplementary Fig. 3E, Additional File 1). The spatial distribution of scarlet fever showed that the high incidence of scarlet fever was distributed in the eastern coastal area and Central Shandong, with a high incidence in Jinan, Zibo, Yantai and Yantai (See Fig. 2F). The result of scarlet fever after Bayesian smoothing was consistent with that of Natural Break classification (See Supplementary Fig. 2F, Additional File 1). The ER of 45 counties was greater than 1 for scarlet fever (See Supplementary Fig. 3F, Additional File 1). The spatial distribution of influenza showed that West Shandong (such as Liaocheng, Dezhou, Heze and Jinan) had higher incidence of influenza than others, and the counties with the top incidences of influenza were Pingyuan County in Dezhou (33.3 per 100,000), Central District in Jining (31.7 per 100,000) and Yucheng County-level city in Dezhou (29.2 per 100,000, Fig. 2G & See Supplementary Fig. 2G, Additional File 1). There were 45 counties in the incidence of influenza with ER above 1 (See Supplementary Fig. 3G, Additional File 1). A higher incidence of mumps was in Binzhou, Jinan and Taian. The top average annual incidences of mumps were in Guangrao County in Dongying (44.3 per 100,000), Shizhong District in Jining (41.6 per 100,000) and Rencheng County in Jining (35.0 per 100,000, Fig. 2H & Supplementary Fig. 2H, Additional File 1). The ER map of mumps showed 59 counties with ER greater than 1 (See Supplementary Fig. 3H, Additional File 1). The spatial distribution of rubella showed that the areas with high incidence of rubella were in northern Shandong and Middle Shandong, such as Binzhou, Dongying, Jinan and Binzhou. The highest average annual incidence of rubella was in Huantai County in Zibo (13.6 per 100,000, Fig. 2I & Supplementary Fig. 2I, Additional File 1). The ER map of rubella showed there were 69 counties with ER above 1. Among them, the ERs of rubella in Changle County, Gaoqing County and Changdao County were higher than other counties (See Supplementary Fig. 3I, Additional File 1).

### Spatial autocorrelation analysis

**Fig. 3:**
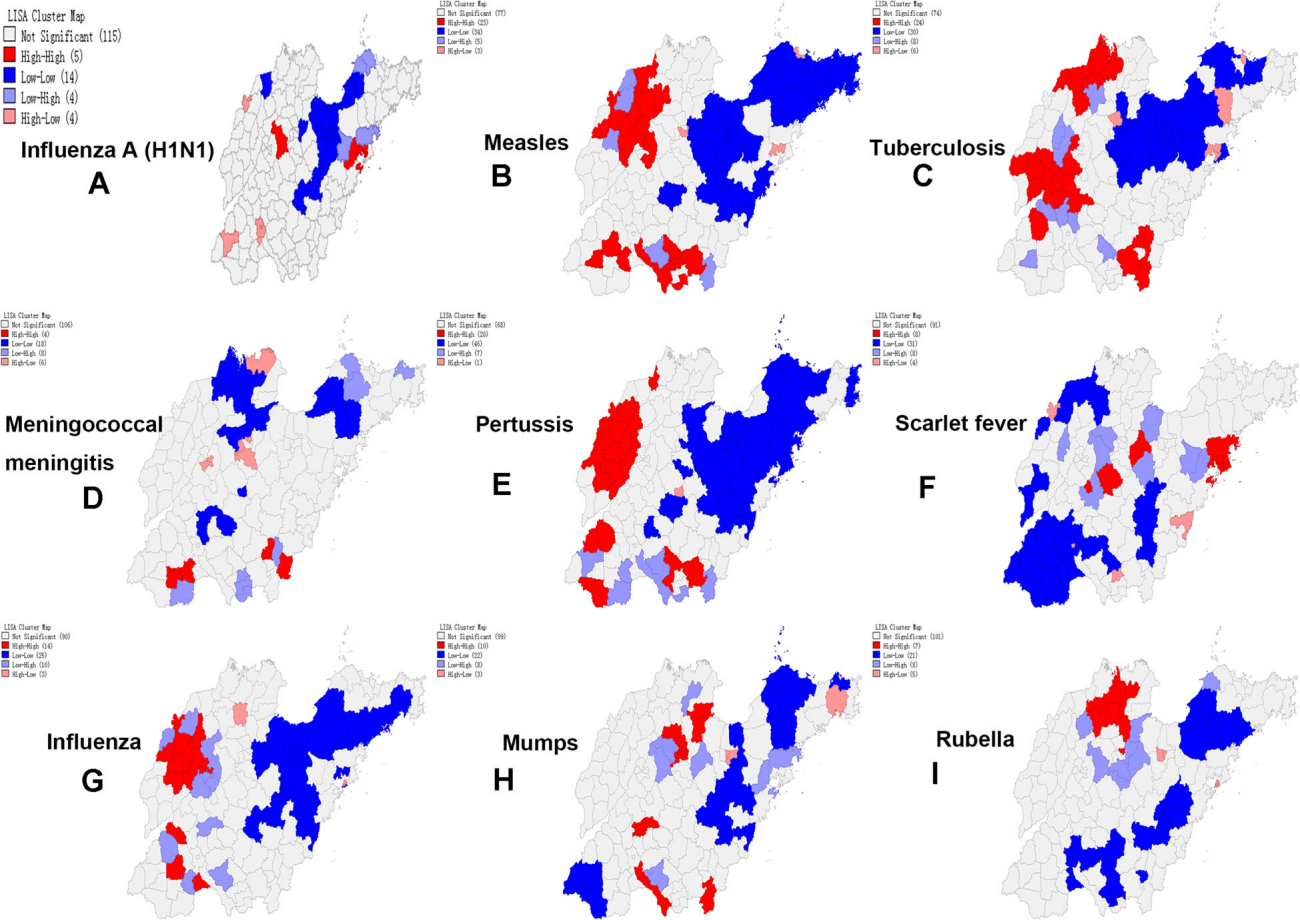
The LISA cluster maps of notifiable respiratory infectious diseases during 2005–2014 in Shandong Province. The Shandong map was created with GeoDa software based on the public geographical data downloaded from Resource and Environment Science and Data Center, Institute of Geographic Sciences and Natural Resources Research, CAS (https://www.resdc.cn/)

The result of global spatial autocorrelation analysis showed that the Moran’s *I* of average annual incidence of each notifiable respiratory infectious disease in Shandong Province ranged from 0.062 to 0.353. This result indicated that there was an evident spatial correlation blinding the cases of notifiable respiratory infectious diseases except meningococcal meningitis (Table 2). Only those counties whose local Moran’s *I* had reached the significance level of 0.05 were presented on the LISA cluster maps. The local spatial autocorrelation analysis showed that the incidence of each notifiable respiratory infectious disease in Shandong Province had an obvious clustering (See Fig. 3). The LISA result of influenza A (H1N1) showed that there were 5 HH cluster regions (i.e., hotspots), which were mainly concentrated in Qingdao and Jinan. While there were 14 LL cluster regions (i.e., coldspots), which were mainly concentrated in Yantai, Weifang and Linyi (See Fig. 3A). As seen in Fig. 3A, Gaomi, Jimo, Longkou and Penglai were LH cluster regions, and municipal district of Heze, municipal district of Jining, municipal district of Dezhou were HL cluster regions. For measles, the 23 HH cluster regions were mainly located in South Shandong and Dezhou, while the 34 LL cluster regions were mainly located in Northeast Shandong (See Fig. 3B). As seen in Fig. 3B, Zaozhuang and Linyi displayed a LH cluster feature, and municipal district of Zibo, Jimo, municipal district of Yantai displayed a HL cluster feature. We identified 24 HH cluster regions for tuberculosis, which were primarily concentrated in Binzhou, Liaocheng and Linyi. While 30 LL cluster regions were identified for tuberculosis, which were primarily concentrated in Weifang, Zibo and Yantai (See Fig. 3C). As seen in Fig. 3C, the LH cluster regions of tuberculosis were scattered in Dezhou, Heze and Jining, and the HL cluster regions were mainly distributed in Yantai. For meningococcal meningitis, we identified 4 HH cluster regions, 18 LL cluster regions, 8 LH cluster regions and 6 HL cluster regions (See Fig. 3D). There were 20 HH cluster regions for pertussis, mainly distributed in North Liaocheng and South Dezhou, Ju County, Juancheng County, Yuncheng County, Cangshan County and Central District of Zaozhuang. While, there were 46 LL cluster regions, mainly distributed in Northeast Shandong. We also identified 7 LH cluster regions and a HL cluster region (See Fig. 3E). The 8 HH cluster regions and 31 LL cluster regions for scarlet fever were mostly from counties in Qingdao, Weifang, Laiwu, South Shandong and Dezhou. Some counties in Qingdao, Weifang, Jinan, Rizhao and Dezhou were identified for LH or HL cluster regions (See Fig. 3F). The LISA result of influenza showed that there were 14 HH cluster regions, which were mainly concentrated in Dezhou and Jining. While there were 25 LL cluster regions, which were mainly concentrated in Yantai, Weifang and Qingdao (See Fig. 3G). And 10 LH cluster regions and 3 HL cluster regions were identified for influenza (See Fig. 3G). For mumps, the 10 HH cluster regions were mainly located in Dongying, while the 22 LL cluster regions were mainly located in Yantai, Weifang, Rizhao (See Fig. 3H). For rubella, we identified 7 HH cluster regions, 21 LL cluster regions, 8 LH cluster regions and 5 HL cluster regions (See Fig. 3I).

**Table 2:** The Moran’s *I* of global spatial autocorrelation analysis for notifiable respiratory infectious diseases in Shandong Province during the study period

Disease	Moran’s *I*	*Z*	*P*-value
Influenza A (H1N1)	0.076	2.040	0.034
Measles	0.353	8.664	0.001
Tuberculosis	0.346	8.278	0.001
Meningococcal meningitis	0.062	1.642	0.061
Pertussis	0.238	6.251	0.001
Scarlet fever	0.157	4.170	0.001
Influenza	0.130	3.691	0.008
Mumps	0.166	4.202	0.001
Rubella	0.093	2.423	0.015

### Spatial-temporal scanning analysis

**Fig. 4:**
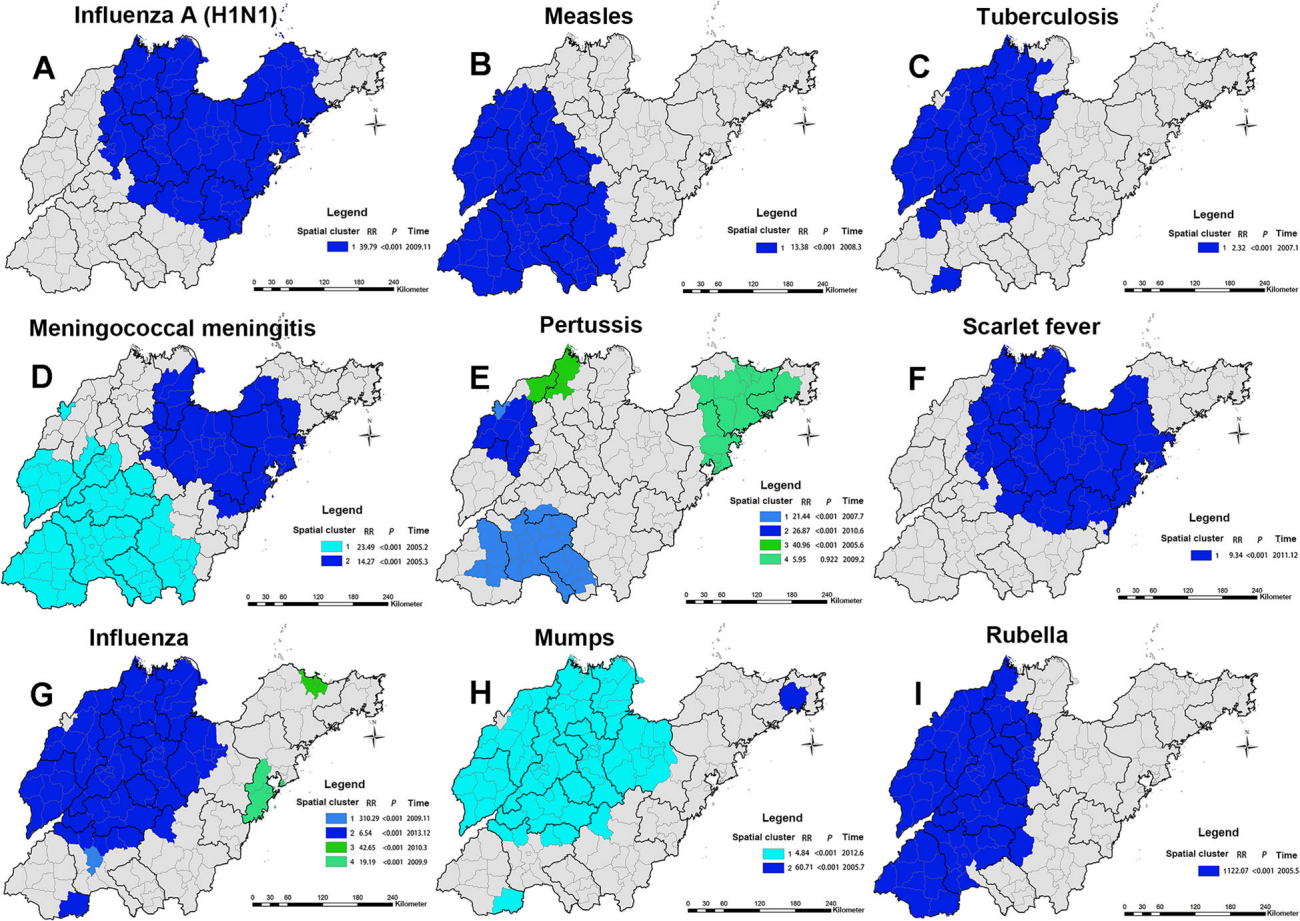
Spatial-temporal clusters of notifiable respiratory infectious diseases during 2005–2014 in Shandong Province. The Shandong map was created with ArcGIS software based on the public geographical data downloaded from Resource and Environment Science and Data Center, Institute of Geographic Sciences and Natural Resources Research, CAS (https://www.resdc.cn/)

Figure 4 and Table S1 (See Supplementary Table 1, Additional File 1) show that notifiable respiratory infectious diseases in Shandong Province had a non-random spatial and temporal distribution. The most likely cluster times for notifiable respiratory infectious diseases were in winter-and-spring during the study period except pertussis and mumps (See Supplementary Table 1, Additional File 1). But the most likely clusters in space were somewhat different for different notifiable respiratory infectious diseases (See Fig. 4).

The most likely cluster time for influenza A (H1N1) was November 2009, and the most likely cluster areas were mainly concentrated in Central Shandong and Eastern Shandong (74 counties in total), such as Zhucheng County-level city, Penglai County-level city, Rongcheng County-level city, Lijin County, municipal district of Weihai, etc. The cluster center was located at 36°77′ N, 119°22′ E, the cluster radius was 199.83 km, the average annual rate within this range was 27.1 per 100,000, and the RR was 39.79 (*P* < 0.001, See Fig. 4A).

The most likely cluster time for measles was March 2008, and the most likely cluster areas were mainly concentrated in Southwest Shandong (66 counties in total), such as Laoling County-level city, Ningjin County, Huimin County, Ling County, Shanghe County, etc. The cluster center was located at 35°28′ N, 115°08′ E, the cluster radius was 292.83 km, the average annual rate within this range was 34.0 per 100,000, and the RR was 13.38 (*P* < 0.001, See Fig. 4B).

The most likely cluster time for tuberculosis was January 2007, and the most likely cluster areas were mainly concentrated in Central Shandong and Northwest Shandong (58 counties in total), such as Wucheng County, Xiajin County, Pingyuan County, Gaotang County, Ling County, etc. The cluster center was located at 37°17′ N, 116°43′ E, the cluster radius was 183.32 km, the average annual rate within this range was 82.1 per 100,000, and the RR was 2.32 (*P* < 0.001, See Fig. 4C).

The most likely cluster time for meningococcal meningitis was February 2005, and the most likely cluster areas were mainly concentrated in Southwest Shandong (56 counties in total), such as municipal district of Dezhou, Qihe County, municipal district of Jinan, Linqing County-level city, Changqing County, etc. The cluster center was located at 34°80′ N, 116°08′ E, the cluster radius was 230.36 km, the average annual rate within this range was 0.5 per 100,000, and the RR was 23.49 (*P* < 0.001, See Fig. 4D). We also obtained one secondary cluster for meningococcal meningitis, which consisted of 37 cluster counties. The secondary spatial-temporal clusters were mainly in Central Shandong and Eastern Shandong (such as Lijin County, Laixi County-level city, Boxing County, Huantai County, municipal district of Zibo, etc.) during March 2005 (See Fig. 4D & Supplementary Table 1, Additional File 1).

The most likely cluster time for pertussis was July 2007, and the most likely cluster areas were mainly concentrated in Southwest Shandong (22 counties in total), such as Yutai County, Jinxiang County, Tengzhou County-level city, etc. The cluster center was located at 35°00′ N, 116°65′ E, the cluster radius was 98.58 km, the average annual rate within this range was 3.5 per 100,000, and the RR was 21.44 (*P* < 0.001, Fig. 4E). We also obtained two secondary clusters for pertussis, which consisted of 12 cluster counties. The secondary spatial-temporal clusters were mainly in Northwest Shandong (such as Pingyuan County, Ling County, Yucheng County-level city, etc.) during June 2010 and June 2005 (See Fig. 4E & Supplementary Table 1, Additional File 1).

The most likely cluster time for scarlet fever was December 2011, and the most likely cluster areas were mainly concentrated in Central Shandong and North Shandong (64 counties in total), such as Zibo, Binzhou, Laiwu, Jinan, etc. The cluster center was located at 36°70′ N, 118°82′ E, the cluster radius was 168.54 km, the average annual rate within this range was 21.2 per 100,000, and the RR was 9.34 (*P* < 0.001, See Fig. 4F).

The most likely cluster time for influenza were November 2009, and the most likely cluster areas were concentrated in Shizhong District and Rencheng District in Jining. The cluster center was located at 35°42′ N, 116°58′ E, the cluster radius was 0 km, the average annual rate within this range was 751.6 per 100,000, and the RR was 310.29 (*P* < 0.001, See Fig. 4G). We also obtained three secondary clusters for influenza, which consisted of 77 cluster counties. The secondary spatial-temporal clusters were mainly in Northwest Shandong, Penglai County-level city, municipal district of Qingdao, Jiaozhou County-level city, etc. during December 2013, March 2010 and September 2009 (See Fig. 4G & Supplementary Table 1, Additional File 1).

The most likely cluster time for mumps was June 2012, and the most likely cluster areas were mainly concentrated in Central Shandong and Northwest Shandong (72 counties in total), such as Lijin County, Qingyun County, Laoling County-level city, Ningjin County, Yangxin County, etc. The cluster center was located at 37°73′ N, 117°23′ E, the cluster radius was 239.96 km, the average annual rate within this range was 51.8 per 100,000, and the RR was 4.84 (*P* < 0.001, See Fig. 4H). We also obtained one secondary cluster for mumps, which consisted of 1 cluster county. And the cluster time was July 2005 (See Fig. 4H & Supplementary Table 1, Additional File 1).

The most likely cluster time for rubella was May 2005, and the most likely cluster areas were mainly concentrated in West Shandong (56 counties in total), such as Xiajin County, Gaotang County, Linqing County-level city, Wucheng County, Pingyuan County, etc. The cluster center was located at 36°85′ N, 115°70′ E, the cluster radius was 230.36 km, the average annual rate within this range was 190.5 per 100,000, and the RR was 122.07 (*P* < 0.001, See Fig. 4I).

## Discussion

This study has, for the first time, revealed the spatial-temporal epidemiology of notifiable respiratory infectious diseases in Shandong Province extensively and systematically using monitoring data. The scan statistical technique can provide means to detect spatiotemporal distribution of notifiable respiratory infectious diseases, as well as to identify high-risk areas. Spatial-temporal analysis of notifiable respiratory infectious diseases in our study is not only helpful to understand the epidemic characteristics of notifiable respiratory infectious diseases in Shandong Province, but also provides scientific basis for health policy makers, public health professionals and clinicians to control and prevent notifiable respiratory infectious diseases.

The results of the time series of notifiable respiratory infectious diseases indicated that the incidence of notifiable respiratory infectious diseases in Shandong Province has obvious seasonality, with peaking in winter and spring and a few notifiable respiratory infectious diseases in early summer, which may be related to climatic factors [25–28]. In addition, we found that the incidence of measles, tuberculosis and meningococcal meningitis showed a downward trend, and the incidence of influenza A (H1N1) and rubella showed sporadic and have an outbreak trend occasionally. However, the incidence of scarlet fever, mumps and influenza had been rising in recent years in Shandong Province, which suggested to improve the awareness of prevention and treatment of notifiable respiratory infectious diseases. The temporal distribution characteristics of notifiable respiratory infectious diseases were consistent with the findings of Mao et al. [17]. The reasons for the increased incidence of these notifiable respiratory infections may be as follows: some notifiable respiratory infectious pathogens mutate, which can increase susceptibility of humans, such as the emergence of new influenza virus [29]; and some notifiable respiratory infections are asymptomatic or subclinical infections, which have the potential to cause outbreaks.

The spatial distribution characteristics of different notifiable respiratory infectious diseases are different. Our study showed that the incidence of notifiable respiratory infectious diseases in Shandong Province is mainly in West Shandong and Central Shandong, followed by South Shandong. The incidence of notifiable respiratory infectious diseases in coastal areas is relatively well controlled, indicating that the distribution characteristics of notifiable respiratory infectious diseases are closely related to geographical and environmental factors [30, 31]. The analysis of this study showed that there is obvious spatial clustering of each notifiable respiratory infectious disease. The hotspots areas of influenza A (H1N1) were mainly in Qingdao and Jinan, the hotspots areas of measles were mainly in South Shandong and Dezhou, the hotspots areas of tuberculosis were mainly in Binzhou, Liaocheng and Linyi, the hotspots areas of meningococcal meningitis were mainly in Heze and Linyi, the hotspots areas of pertussis are mainly in North Liaocheng, South Dezhou, Ju County, Juancheng County, Yuncheng County, Cangshan County and Central District of Zaozhuang, the hotspots areas of scarlet fever were mainly in Qingdao, Laiwu and Qingzhou County-level city, the hotspots areas of influenza were mainly in Dezhou and Jining, the hotspots areas of mumps was mainly in Dongying, and the hotspots areas of rubella was mainly in Bingzhou. The high-risk areas have been identified for some notifiable respiratory infectious diseases in Shandong Province in several studies, for example, measles [32, 33], tuberculosis [34], pertussis [35], and mumps [36]. And the results of these studies are similar to our findings. The transmission of various notifiable respiratory infectious diseases is affected by different factors, so different preventives strategies should be taken for different notifiable respiratory infectious diseases. For example, in view of influenza, comprehensive preventive measures should be taken, including strengthening surveillance and immunization prevention. We should strengthen the development of a national influenza surveillance network, improve the quality of the work, formulate a national influenza vaccine immunization guidance plan, and ensure the immunization of key groups. For tuberculosis, professional institutions for the prevention and control of tuberculosis at all levels should strengthen the cooperation with health care units while earnestly doing a good job in the detection patients who visit the hospital due to symptoms, and systematically adopt the recommendation method for patients to improve the detection rate of patients. We also should implement the examination of key populations in areas with high prevalence of tuberculosis, as well as the examination of groups with high incidence such as groups or populations with outbreak of tuberculosis and the regular examination of subjects in key industries.

The temporal-spatial clustering analysis identified the most likely cluster and several secondary clusters for each notifiable respiratory infectious disease, indicating that the incidence of notifiable respiratory infectious diseases is increasing. And the time windows were mainly concentrated in winter-and-spring. The most likely cluster and several secondary clusters for notifiable respiratory infectious diseases differ with the hotspot analysis, because the hotspot analysis did not consider the time factor and arbitrarily conducted static scale selection. The spatial-temporal scanning method achieved effective temporal and spatial integration, which was able to evaluate the clustering in different time windows to achieve a dynamic, three-dimensional and multi-scale analysis [37]. For influenza A (H1N1), especially in Central Shandong and Eastern Shandong, local public-health authority should simultaneously strengthen influenza A (H1N1) surveillance and prevention among high-risk groups. For measles, especially in the 66 counties of Southwest Shandong, local public-health authority should simultaneously strengthen measles surveillance and the coverage rate of measles vaccine in floating population. For tuberculosis, especially in Central Shandong and Northwest Shandong, local public-health authority should simultaneously strengthen health promotion for tuberculosis and surveillance in the high-risk population. For meningococcal meningitis, especially in the 56 counties of Southwest Shandong and 37 counties of Central Shandong and Eastern Shandong, local public-health authority should simultaneously strengthen meningococcal meningitis surveillance and improve the coverage of meningococcal meningitis vaccine in densely populated areas. For pertussis, especially in 22 counties of Southwest Shandong and 12 counties of Northwest Shandong, local public-health authority should simultaneously strengthen pertussis surveillance and the coverage of diphtheria-pertussis-tetanus vaccine among high-risk groups. For scarlet fever, especially in Central Shandong and North Shandong, the health department should strengthen the monitoring and prevention of scarlet fever in high-risk groups and peak periods of scarlet fever. For influenza, especially in Northwest Shandong and Shizhong District and Rencheng District in Jining, the health department should strengthen the monitoring and health publicity and education among influenza season. For mumps, especially in Central Shandong and Northwest Shandong, the health department should simultaneously strengthen mumps surveillance and prevention among high-risk population. For rubella, especially in 56 counties of West Shandong, the health department should simultaneously strengthen rubella surveillance and health promotion among high-risk population.

Several limitations of this study should be acknowledged. First, the spatial-temporal scanning was very useful to explore spatially aggregated data and to highlight the riskiest areas to conduct more accurate analysis [38]. But we must note that ecologic bias was inevitable in any ecological study [39]. For example, we took counties as the analysis space unit in this study to conduct the spatial-temporal cluster of notifiable respiratory infectious diseases. The results could only reflect the aggregation of the county, the urban and rural distribution characteristics of notifiable respiratory infectious diseases in Shandong Province could not be inferred. Second, local Moran’s *I* could reflect the clustering of notifiable respiratory infectious diseases in spatial pattern, but it might be disturbed by population fluctuation. And some irregular counties could limit the power of circular scan statistics. Third, because Shandong Center for Disease Control and Prevention only shared with us the data from 2005 to 2014, we failed to analyze the temporal and spatial distributions of notifiable respiratory infectious diseases from 2015 to 2019, which might be slightly different from 2014 to 2019. Finally, this study did not evaluate potential environmental risk factors and population characteristics associated with the different temporal and spatial distributions of notifiable respiratory infectious diseases. Therefore, it is important to conduct further studies to identify the geographical risk factors and determine precise local prevention and control measures.

## Conclusions

In summary, our study revealed that different notifiable respiratory infectious diseases display different trends. Our study confirmed the spatial autocorrelation of notifiable respiratory infectious diseases, and spatial-temporal clusters with high risk of notifiable respiratory infectious diseases in Shandong Province. Different notifiable respiratory infectious diseases had different hotspots and temporal and spatial clusters. Using this information, decision makers may wish to implement targeted interventions to control and prevent notifiable respiratory infectious diseases in areas of high transmission. Meanwhile, we hope these findings help to point out the direction of future research that contribute to notifiable respiratory infectious diseases control in Shandong Province, and provide useful information for public health officials to develop targeted prevention and preparedness measures for notifiable respiratory infectious diseases.

## Supplementary Information

**Additional file 1.** Supplementary a table and three figures.

## Data Availability

Shandong CDC did not provide consent for sharing its data publicly. Data are available from the Shandong First Medical University & Shandong Academy of Medical Sciences for researchers who meet the criteria for access to confidential data. Please address reasonable requests to Dr. Guoyong Ding at dgy-153@163.com.

## **Abbreviations**

CI: Confidence interval
DALYs: Disability adjusted life years
ER: Excess risk
GBD: Global Burden of Disease
GIS: Geographic information systems
HH: High-incidence regions surrounded by high-incidence regions
HL: High-incidence regions surrounded by low-incidence regions
LH: Low-incidence regions surrounded by high-incidence regions
LISA: Local indicators of spatial association
LL: Low-incidence regions surrounded by low-incidence regions
LLR: Log-likelihood ratio
RR: Relative risk
RSV: Respiratory syncytial virus
SARS: Severe acute respiratory syndrome
