# Supplementary material for: Analysis of spatial-temporal distribution of notifiable respiratory infectious diseases in Shandong Province, China during 2005–2014
Source: BMC Public Health. 2021 Aug 30;21:1597. doi: 10.1186/s12889-021-11627-6 (PMC8403828; doi:10.1186/s12889-021-11627-6)

**SUPPLEMENTARY INFORMATION**

**Analysis of spatial-temporal distribution of respiratory infectious diseases in Shandong Province, China during 2005-2014**

Xiaomei Li^1,2#^, Dongzhen Chen^1#^, Yan Zhang^3#^, Xiaojia Xue^4^, Shengyang Zhang^5^, Meng Chen^6^, Xuena Liu^1*^, Guoyong Ding^1*^

^1^ School of Public Health, Shandong First Medical University & Shandong Academy of Medical Sciences, 271016, Taian, Shandong Province, China

^2^ Liaocheng Center for Disease Control and Prevention, 252100, Liaocheng, Shandong Province, China

^3^ Guiqian International General Hospital, 550018, Guiyang, Guizhou Province, China

^4^ Qingdao Municipal Center for Disease Control & Prevention, 266033, Qingdao, Shandong Province, China

^4^ Shandong Center for Disease control and Prevention, 250014, Jinan, Shandong Province, China

^6^ Jining Center for Disease Control and Prevention, 272113, Qingdao, Shandong Province, China

^#^These authors contributed equally for this work.

^*^Corresponding Authors (Guoyong Ding and Xuena Liu)

**Table S1.** Spatial-temporal cluster analysis for respiratory infectious diseases in Shandong Province during 2005-2014.

**Fig. S1**. Location of the study areas in China. The China and Shandong maps were created with ArcGIS software based on the public geographical data downloaded from Resource and Environment Science and Data Center, Institute of Geographic Sciences and Natural Resources Research, CAS (https://www.resdc.cn/).

**Fig. S2**. The average annual incidence rate of respiratory infectious diseases by the spatial Bayesian smoothing method from 2005-2014 in Shandong Province. The Shandong map was created with GeoDa software based on the public geographical data downloaded from Resource and Environment Science and Data Center, Institute of Geographic Sciences and Natural Resources Research, CAS (https://www.resdc.cn/).

**Fig. S3**. The excess risk map of the average annual incidence rate of respiratory infectious diseases from 2005-2014 in Shandong Province. The Shandong map was created with GeoDa software based on the public geographical data downloaded from Resource and Environment Science and Data Center, Institute of Geographic Sciences and Natural Resources Research, CAS (https://www.resdc.cn/).

**Table S1.** Spatial-temporal cluster analysis for respiratory infectious diseases in Shandong Province during 2005-2014.

| Disease | Cluster grade^*^ | Number of counties | Number of cases | Expected cases | RR | LLR | *P*-value | Cluster time |
| --- | --- | --- | --- | --- | --- | --- | --- | --- |
| Influenza A (H1N1) | 1 | 74 | 1037 | 33.12 | 39.79 | 2682.975 | <0.001 | Nov-2009 |
| Measles | 1 | 66 | 1367 | 107.45 | 13.38 | 2248.567 | <0.001 | Mar-2008 |
| Tuberculosis | 1 | 58 | 2501 | 1080.89 | 2.32 | 680.954 | <0.001 | Jan-2007 |
| Meningococcal meningitis | 1 | 56 | 18 | 0.83 | 23.49 | 38.933 | <0.001 | Feb-2005 |
|  | 2 | 37 | 7 | 0.50 | 14.27 | 12.007 | 0.022 | Mar-2005 |
| Pertussis | 1 | 22 | 45 | 2.16 | 21.44 | 94.462 | <0.001 | Jul-2007 |
|  | 2 | 8 | 15 | 0.56 | 26.87 | 34.857 | <0.001 | Jun-2010 |
|  | 3 | 4 | 10 | 0.25 | 40.96 | 27.339 | <0.001 | Jun-2005 |
|  | 4 | 19 | 8 | 1.35 | 5.95 | 7.601 | 0.922 | Feb-2009 |
| Scarlet fever | 1 | 64 | 787 | 86.92 | 9.34 | 1044.860 | <0.001 | Dec-2011 |
| Influenza | 1 | 2 | 679 | 2.25 | 310.29 | 3209.269 | <0.001 | Nov-2009 |
|  | 2 | 67 | 619 | 96.79 | 6.54 | 632.102 | <0.001 | Dec-2013 |
|  | 3 | 4 | 122 | 2.87 | 42.65 | 338.437 | <0.001 | Mar-2010 |
|  | 4 | 6 | 132 | 6.91 | 19.19 | 264.539 | <0.001 | Sep-2009 |
| Mumps | 1 | 72 | 1967 | 412.34 | 4.84 | 1530.240 | <0.001 | Jun-2012 |
|  | 2 | 1 | 378 | 6.25 | 60.71 | 1179.675 | <0.001 | Jul-2005 |
| Rubella | 1 | 56 | 7559 | 92.71 | 122.07 | 27206.694 | <0.001 | May-2005 |

RR, relative risk; LLR, log likelihood ratio. ^*^The most likely cluster, first secondary cluster, second secondary cluster, and third secondary cluster were represented by 1, 2, 3 and 4, respectively.

**Fig. S1**. Location of the study areas in China. The China and Shandong maps were created with ArcGIS software based on the public geographical data downloaded from Resource and Environment Science and Data Center, Institute of Geographic Sciences and Natural Resources Research, CAS (https://www.resdc.cn/).


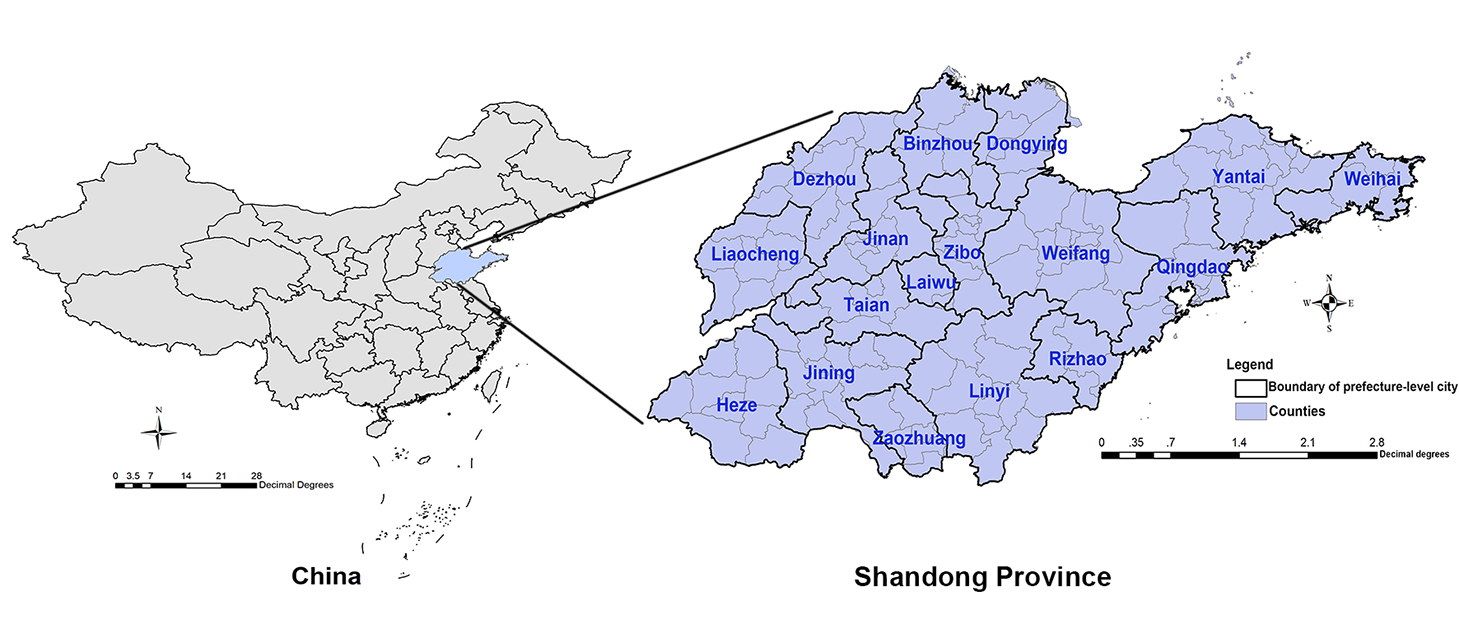


**Fig. S2**. The average annual incidence rate of respiratory infectious diseases by the spatial Bayesian smoothing method from 2005-2014 in Shandong Province. The Shandong map was created with GeoDa software based on the public geographical data downloaded from Resource and Environment Science and Data Center, Institute of Geographic Sciences and Natural Resources Research, CAS (https://www.resdc.cn/).


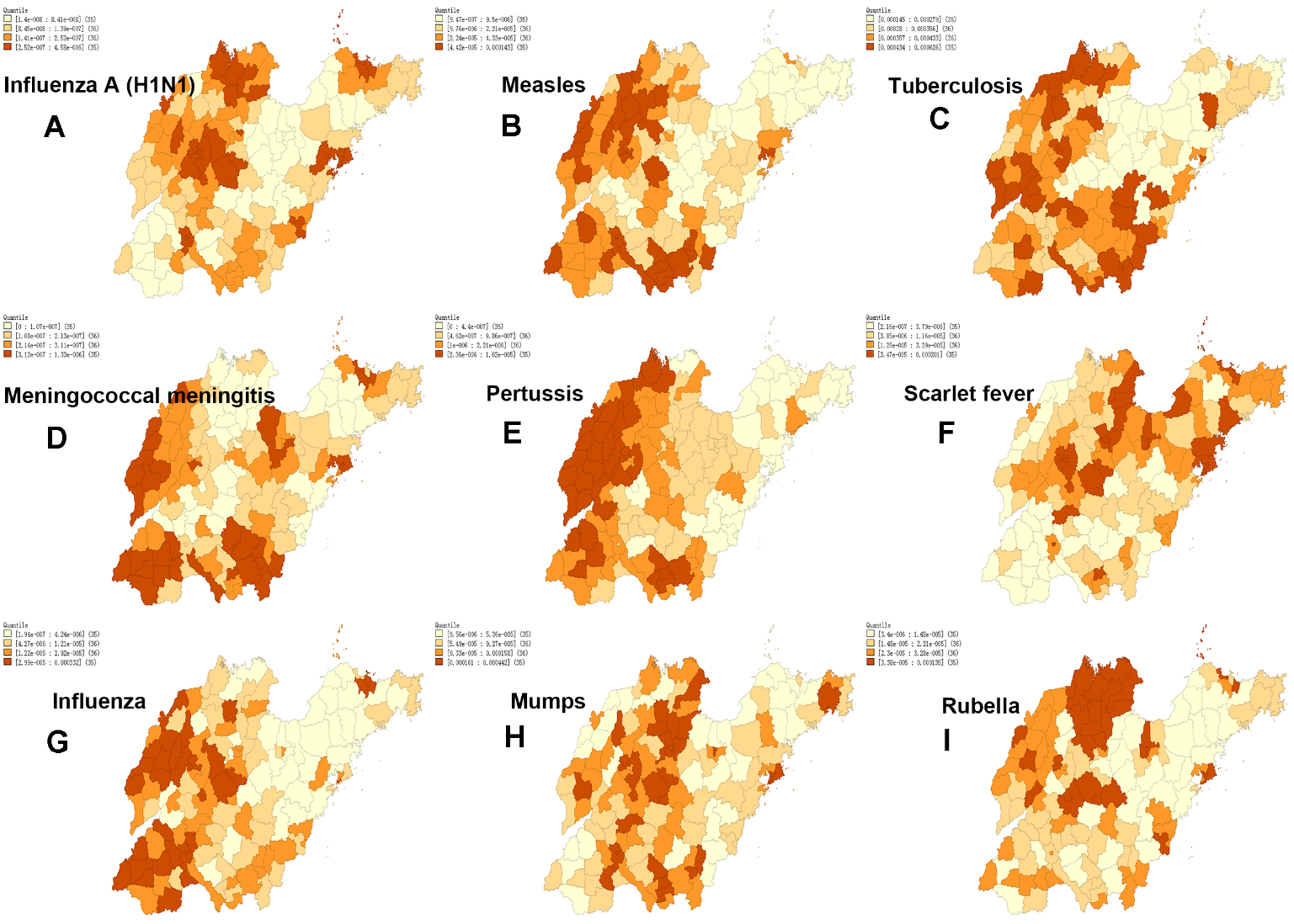


**Fig. S3**. The excess risk map of the average annual incidence rate of respiratory infectious diseases from 2005-2014 in Shandong Province. The Shandong map was created with GeoDa software based on the public geographical data downloaded from Resource and Environment Science and Data Center, Institute of Geographic Sciences and Natural Resources Research, CAS (https://www.resdc.cn/).


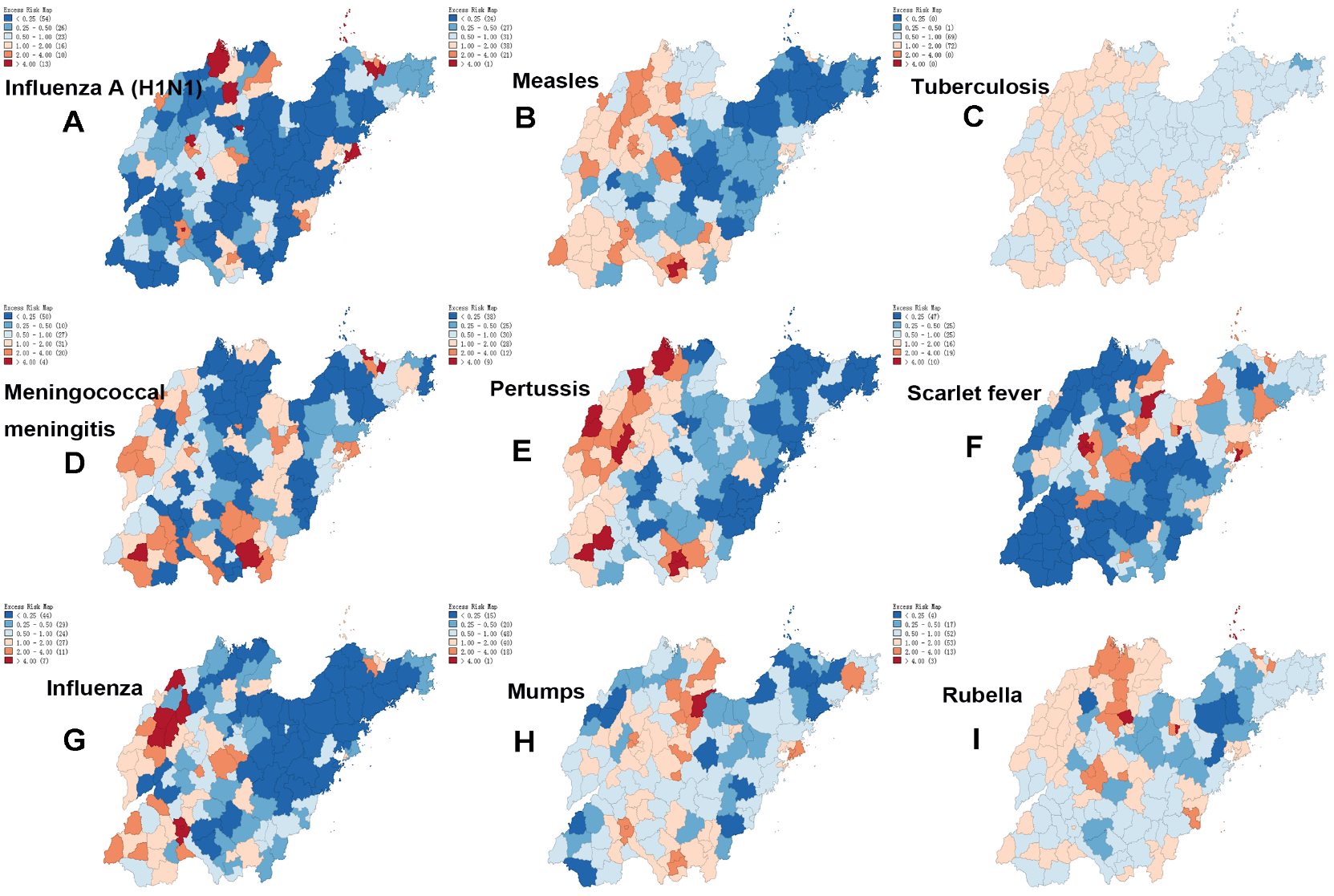

Supplement: Supplementary file 1 — Additional file 1. Supplementary a table and three figures. [file 12889_2021_11627_MOESM1_ESM.docx]
